# The impact of isolation on comorbidity of PTSD symptoms and depression: evidence from PTRP-5-6 in China

**DOI:** 10.1186/s12889-023-17450-5

**Published:** 2024-01-02

**Authors:** Wenjie Yan, Zhilei Shang, Lili Wu, Hongli Lv, Yanpu Jia, Jingye Zhan, Jing Wang, Hui Ouyang, Weizhi Liu, Wenfang Chen

**Affiliations:** 1 Lab for Post-traumatic Stress Disorder, Faculty of Psychology and Mental Health, Naval Medical University, 800 Xiangyin Road, Shanghai, 200433 China; 2 Department of Urology, Jinling Hospital, Clinical School of Medical College, Nanjing University, 305 East Zhongshan Road, Nanjing, 210000 P. R. China; 3 Department of Gastroenterology and Hepatology, Jinling Hospital, Clinical School of Medical College, Nanjing University, Nanjing, 210000 P. R. China; 4 The Emotion & Cognition Lab, Faculty of Psychology and Mental Health, Naval Medical University, Shanghai, 200433 China

**Keywords:** PTSS, Depressive symptoms, Comorbidity, PTRP

## Abstract

**Background:**

The Omicron pandemic struck Shanghai, China, resulting in impairments of both physical and psychological health on those patients who were confirmed and transferred to the Fangcang shelters. The way of isolation led to high risk of posttraumatic stress symptoms (PTSS) and depressive symptoms among the patients in Fangcang shelters. We aim to estimate the prevalence and comorbidity of PTSS and depressive symptoms in patients from China’s Fangcang shelters during the epidemic.

**Methods:**

Demographic information questionnaire, the posttraumatic stress disorder checklist for DSM-5 (PCL-5), and Patient Health Questionnaire (PHQ-9) were used in the study. The data were collected online via mobile phones during 10th April to 20th April, 2022, as part of our Psychological Trauma Recover Project-5-6 (PTRP-5-6), a longitudinal study focusing on individuals who have experienced trauma.

**Results:**

A total of 336 subjects were included in the analysis. The results revealed (1) the prevalence of depressive symptoms, and PTSS were 30.1% (cut-off = 10) and 6% (cut-off = 33); (2) Multiple logistic regression showed that female (OR = 3.04, *p* < 0.05), suffering from dyspnea (OR = 5.83, *p* < 0.05) or gastrointestinal symptoms (OR = 6.38, *p* < 0.05) were risk factors for PTSS; higher education level (OR = 3.27, *p* < 0.05) and suffering from dizziness or headache (OR = 2.46, *p* < 0.05) were risk factors for depressive symptoms; (3)Respectively, 85% of the patients who reported PTSS also experienced depressive symptoms, 16.8% of the patients who reported depressive symptoms presented PTSS.

**Conclusion:**

In the context of COVID-19, the comorbidity rate of PTSS and depressive symptoms among patients in Fangcang shelters increased with the severity of depressive symptoms.

## Background

The coronavirus disease 2019 (COVID-19) caused by severe acute respiratory syndrome-2(SARS-CoV-2) virus, has induced 608 million confirmed cases and 6.5 million deaths By September 2022, leading to long-term health burden and mental sequelae for the public [1]. Obviously, it has been considered as a global-concerning infectious emergency. The B.1.1.529 variant of virus was designated by WHO as Omicron on 26 November 2021, caused a surge of infections four months later in Shanghai, China [2]. Public vehicles like buses, subways, and online car-hailing services are suspended in locked-down regions [3]. Since then, the municipal government took a series of measures to mitigate the spread of the virus, such as building shelter hospitals to accommodate the increasing patients and conducting rounds of nucleic acid testing for residents. The epidemic knocked down the metropolis, constantly threatening public physical and mental health. Patients who tested positive were transferred to the shelters without adequate preparations, causing significant disruption among the majority of the public due to quarantine measures and restrictions that disrupted their daily routines.

Several studies have provided evidence for the COVID-19 related mental distress among individuals [4, 5]. Definitely, posttraumatic stress disorder (PTSD) and depression were two kinds of common mental distress [6].Relevant studies have shown a high incidence of depression in patients, as well as among frontline health workers [7, 8]. Furthermore, in the systematic meta-analysis of 15,538 individuals affected by the public health emergencies (PHEs), the estimated prevalence (17%) of post-traumatic stress syndromes during the last two decades indicated that post-traumatic stress symptoms frequently occur as a result of PHEs. [9]. Investigations conducted on survivors of public health emergencies such asCOVID-19,the Middle East Respiratory Syndrome (MERS) infection or severe acute respiratory syndrome (SARS) pandemic in 2003, have indicated the presence of prolonged psychiatry sequela for a minimum duration of six months following the pandemic [10–12].

It is important to highlight that posttraumatic stress disorder and depression exhibit a strong correlation as comorbid conditions. Depressive symptoms may manifest in isolation or co-occur with PTSD following trauma exposure (TE) [13]. Evidence has demonstrated that the presence of comorbidity is indicative of an elevated risk for suicide [14]. The comorbidity of these two conditions may give rise to more severe consequence such as significant increased risk of suicidal behaviors compared to individuals with a single diagnosis [14, 15]. A considerable portion of studies have respectively reported the prevalence of PTSD and depression during the outbreak in patients who were confirmed, while limited attention has been given to exploring the presence of comorbidity between the disorders. Half of the survivors recovered from COVID-19 still bear a heavy physical burden or psychiatric sequelae for at least 12 months [16]. To set the stage for later outcomes and crucially inform interventions, immediate research priorities are to monitor and report rates of depression, PTSD, suicide, and other mental health issues. In this way, the current study aims to assess the psychological status of patients in Fangcang shelters and investigate the comorbidity between PTSD and depression.

## Methods

### Study design and participants

The data was obtained from part of the psychological trauma recover project (PTRP) which started on October 1,2019 in China. The PTRP-5-6 was a comprehensive longitudinal project conducted by the Lab for Post-traumatic Stress Disorder of Naval Medical University, focusing on the population experiencing major trauma at the same period of time to answer “Why only a proportion of people experiencing trauma finally develop PTSD” [17]. The data in this survey were collected face-to-face by psychological or health care workers from three Fangcang shelters in Shanghai from April 10, 2022 to April 20, 2022(period coincided with a severe impact of the Omicron outbreak on the city).To minimize physical contact and prevent virus transmission, data collection was conducted through network platform via mobile phone. The inclusion criteria were: 1) patients who confirmed of COVID-19; 2) individuals who were quarantined in the Fangcang shelters; 3) age ≥ 18 years old; 4) without cognitive impairment; 5) voluntary participation. Exclusion criteria were:1) refused to participate; 2) incomplete content and information; 3) only one members’ answer was imported among the same family; 4) history of serious mental illness. Participants who had difficulty in communication and reading, as well as those who failed to complete tasks independently, were classified as having severe cognitive impairment. The assessment of past medical history primarily relied on self-reported information. We obtained the electronic informed written consent from the all the participants and the research was approved by the Committee on Ethics of Medicine at Navy Medical University.

A total of 407 patients were collected information during the survey. Participants who responded less than 2 min or more than 30 min (21) were excluded to improve the quality of questionnaire. Moreover, those who failed to meet the age threshold (13) or submitted invalid questionnaire (37) such as all the same choices were also rejected. In all, 336 valid questionnaires were brought into study.

### Measures

#### Demographic information

All participants were required to complete demographic information (age, sex, education level, and admission information). Additionally, several questions associated with the daily life in shelters were involved.

#### PTSS

The PTSD Checklist for DSM-5(PCL-5) was used to measure the degree of PTSD symptoms [18]. The scale involves 20-items and each item is scored on a 5-point Likert scale rating from 0 (not at all) to 4 (extremely), representing the severity of an individual has been disturbed by PTSD-related symptoms during the past month. The 20 items could be divided into four symptom clusters: intrusive experience (Item 1–5, criterion B), constant avoidance (Item 6–7, criterion C,), negative mood and cognitions (Item 8–14, criterion D), and significant alterations in arousal and reactivity (Item 15–20, criterion E) [19]. According to the previous studies, a cut-off of 33 scores was valid to identify the occurrence of PTSS [19].

#### Depression

The Patient Health Questionnaire-9 (PHQ-9) was applied to assess depression severity in the last two weeks [20]. The questionnaire included nine items and each item was scored on a point ranging from 0 (not at all) to 3 (nearly every day). Setting 10 as cut-off points was reported to have high clinical sensitivity and specificity in depression [21].

### Statistical analysis

Statistical analysis was performed using Statistical Product and Service Solutions (SPSS) 21.0 for Windows. Descriptive statistical analysis was used to characterize demographic sociological factors and clinical characteristics. Chi-square test was used to analyze the correlation between PTSS, depressive symptoms and demographic sociological factors. Pearson bivariate correlation was used to calculate the correlation coefficient between the score of PHQ- 9 and the total score of PCL-5 and the score of each dimension. Multivariate logistic regression was used to analyze the risk factors of PTSS and depressive symptoms.

## Results

### Sociodemographic characteristics

A total of 336 patients were included in the statistical analysis, 75% of whom were males. The youngest patient was 18 years old and the oldest was 67 years old, with an average age of 35.92 ± 11.73 years. Among them, 26.2% reported having family members infected with COVID-19. The most common symptoms of COVID-19 were dry throat, sore throat, cough and fever. Descriptive statistics of sociodemographic data and clinical characteristics are presented in Table 1.

**Table 1:** Socio-demographic and clinical characteristics of 336 patients with COVID-19 and the association with PTSD and depressive symptoms

	Total (n = 336)		With PTSD symptoms		*p*-value		With Depressive symptoms		*p*-value
	N	%	N	%			N	%	
**Socio-demographic characteristics**									
**Gender**									
Male	252	75.0	10	4.0	0.008**		69	27.4	0.064
Female	84	25.0	10	11.9			32	38.1	
**Age**									
18–24	53	15.8	3	5.7	0.746		22	41.5	0.118
25–44	193	57.4	13	6.7			56	29.0	
≥ 45	90	26.8	4	4.4			23	25.6	
**BMI, Kg/m2**									
≤ 18.5	16	4.8	1	6.3	0.852		6	37.5	0.085
18.5–24.9	207	61.8	11	5.3			70	33.8	
25-29.9	92	27.5	6	6.5			21	22.8	
≥ 30	20	6.0	2	10.0			3	15.0	
**Marital status**									
Single	132	39.3	6	4.5	0.312		47	35.6	0.196
Married	187	55.7	14	7.5			49	26.2	
Others	17	5.1	0	0.0			5	29.4	
**Educational level**									
Below undergraduate	270	80.4	17	6.3	0.819		75	27.8	0.044*
Undergraduate	50	14.9	2	4.0			17	34.0	
Master or above	16	4.8	1	6.3			9	56.3	
**Family member infected with COVID-19**									
No	248	73.8	11	4.4	0.049*		74	29.8	0.882
Yes	88	26.2	9	10.2			27	30.7	
**Clinical characteristics**									
**Symptoms at onset**									
Sore throat									
No	209	62.2	13	6.2	0.790		50	23.9	0.002**
Yes	127	37.8	7	5.5			51	40.2	
Dry throat									
No	171	50.9	9	5.3	0.587		41	24.0	0.882
Yes	165	49.1	11	6.7			60	36.4	
Cough									
No	137	40.8	8	5.8	0.942		29	21.2	0.003**
Yes	199	59.2	12	6.0			72	36.2	
Dyspnea									
No	315	93.8	17	5.4	0.096		91	28.9	0.070
Yes	21	6.3	3	14.3			10	47.6	
Fever									
No	240	71.4	15	6.3	0.715		59	24.6	0.001**
Yes	96	28.6	5	5.2			42	43.8	
Fatigue									
No	240	71.4	15	6.3	0.715		56	23.3	< 0.001***
Yes	96	28.6	5	5.2			45	46.9	
Dizziness or Headache									
No	247	73.5	15	6.1	0.876		55	22.3	< 0.001***
Yes	89	26.5	5	5.6			46	51.7	
Gastrointestinal symptoms									
No	302	89.9	15	5.0	0.023*		82	27.2	0.001***
Yes	34	10.1	5	14.7			19	55.9	
Loss of appetite									
No	276	82.1	14	5.1	0.144		70	25.4	< 0.001***
Yes	60	17.9	6	10.0			31	51.7	
Loss of taste or dysgeusia									
No	308	91.7	19	6.2	0.578		89	28.9	0.123
Yes	28	8.3	1	3.6			12	42.9	
Loss of smell or dysosmia									
No	310	92.3	19	6.1	0.637		89	28.7	0.062
Yes	26	7.7	1	3.8			12	46.2	
Hair loss									
No	311	92.6	18	5.8	0.653		94	30.2	0.815
Yes	25	7.4	2	8.0			7	28.0	
Others									
No	322	95.8	17	5.3	0.012*		95	29.5	0.286
Yes	14	4.2	3	21.4			6	42.9	
**Underlying disease**									
Hypertension									
No	305	90.8	17	5.6	0.358		90	29.5	0.489
Yes	31	9.2	3	9.7			11	35.5	
Diabetes									
No	329	97.9	20	6.1	0.501		98	29.8	0.456
Yes	7	2.1	0	0.0			3	42.9	
COPD									
No	332	98.8	20	6.0	0.613		98	29.5	0.155
Yes	4	1.2	0	0.0			3	75.0	
Coronary disease									
No	327	97.3	20	6.1	0.444		98	30.0	0.828
Yes	9	2.7	0	0.0			3	33.3	
Others									
No	323	96.1	20	6.2	0.355		96	29.7	0.500
Yes	13	3.9	0	0.0			5	38.5	

Note: *P*-value were derived from the chi-square test of the variable and PTSD /depressive symptoms.*, *p*-value < 0.05; **, *p*-value < 0.01; ***, *p*-value < 0.001

### Incidence of PTSD and depressive symptoms

A total score of PCL-5 higher than 33 was considered positive for PTSS, and 6% of the participants were positive in PTSS. Chi-square test showed differences in the incidence of PTSS in socio-demographic factors and clinical characteristics in Table 1. Female, having family members infected with COVID-19, suffering from gastrointestinal symptoms had a higher incidence of PTSS.

30.1% of participants exhibited varying degrees of depressive symptoms as assessed by PHQ-9, ranging from mild to severe. The prevalence rates for mild, moderate, moderately severe, and severe depressive symptoms were 19.3%, 6.3%, 2.4%, and 2.1%, respectively. Chi-square analysis showed differences in the incidence of depressive symptoms in sociodemographic factors and clinical characteristics in Table 1. Patients with higher levels of education, or suffering from sore throat, cough, fever, fatigue, dizziness or headache, gastrointestinal symptoms, loss of appetite reported more depressive symptoms.

### Correlations and comorbidities of PTSD and depressive symptoms

Pearson bivariate correlation analysis showed that the PHQ-9 total score was significantly positively correlated with the PCL-5 total score and the scores of each dimension (see Table 2). The correlation coefficients between PHQ-9 total score and PCL-5 total score, B score, C score, D score and E score were 0.599, 0.454, 0.348, 0.502 and 0.609 respectively (P < 0.01).

**Table 2:** Correlation between PHQ-9 and PCL-5 scores

	Mean	SD	1	2	3	4	5	6
**1.PHQ-9 Total score**	3.78	4.85	1.000					
**2.PCL-5 Criterion B**	3.05	3.53	0.454^**^	1.000				
**3.PCL-5 Criterion C**	1.43	1.98	0.348^**^	0.612^**^	1.000			
**4.PCL-5 Criterion D**	3.87	4.31	0.502^**^	0.531^**^	0.480^**^	1.000		
**5.PCL-5 Criterion E**	3.48	3.90	0.609^**^	0.481^**^	0.409^**^	0.648^**^	1.000	
**6.PCL-5 Total score**	11.82	11.70	0.599^**^	0.761^**^	0.682^**^	0.856^**^	0.823^**^	1.000

Note: Bivariate Pearson’s correlation coefficient. *, *p*-value < 0.05; **, *p*-value < 0.01; ***, *p*-value < 0.001

**Fig. 1:**
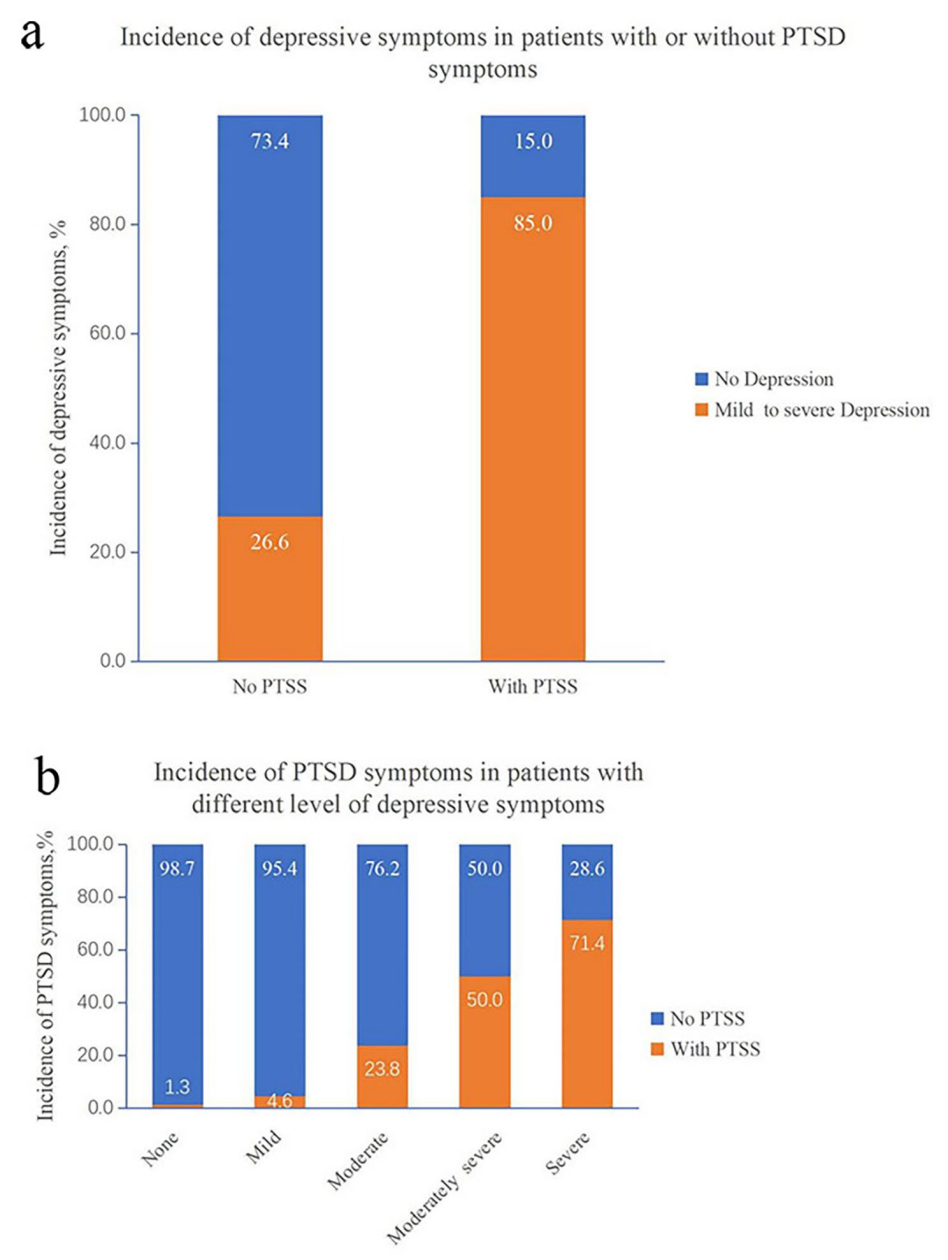
Comorbidity rates of depressive and PTSD symptoms. (**a**) Incidence of depressive symptoms in patients with or without PTSD symptoms. (**b**) Incidence of PTSD symptoms in patients with different level of depressive symptoms

85% of the patients who reported PTSS also reported depressive symptoms (see Fig. 1a), while 16.8% of the patients who exhibited depressive symptoms were also positive for PTSS (see Table 3). The comorbidity rate of PTSS and depressive symptoms increased with the severity of depressive symptoms. The incidences of PTSS in patients with mild, moderate, moderately severe, and severe depressive symptoms were 4.6%, 23.8%, 50.0%, and 71.4%, respectively (see Table 3; Fig. 1b).

**Table 3:** The rate of different severities of depressive symptoms and comorbidity with PTSD symptoms

	Depressive symptoms		Comorbidity with PTSD symptoms		
	N	%	N	No PTSS (%)	With PTSS(%)
**None**	235	69.9	3	98.7	1.3
**Mild**	65	19.3	3	95.4	4.6
**Moderate**	21	6.3	5	76.2	23.8
**Moderately severe**	8	2.4	4	50.0	50.0
**Severe**	7	2.1	5	28.6	71.4
**Mild to severe**	101	30.1	17	83.2	16.8

### Factors influencing PTSD and depressive symptoms

Multiple logistic regression showed that female (OR = 3.04, *p* < 0.05), suffering from dyspnea (OR = 5.83, *p* < 0.05) or gastrointestinal symptoms (OR = 6.38, *p* < 0.05) were potentially risk factors for PTSS after controlling for age, BMI, marital status, education level, whether family members were infected with COVID-19 and clinical symptoms. Higher education level (Master or above vs. Below undergraduate, OR = 3.27, *p* < 0.05) and suffering from dizziness or headache (OR = 2.46, *p* < 0.05) were potentially risk factors for depressive symptoms after controlling other variables(see Tables 4 and 5).

**Table 4:** Multivariable logistic regression to investigate the association between socio-demographic characteristics and PTSD /depressive symptoms

	PTSD symptoms				Depressive symptoms			
	OR	**95%CI**		***p*** **-value**	OR	**95%CI**		***p*** **-value**
		***Lower***	***Upper***			***Lower***	***Upper***	
**Socio-demographic characteristics**								
**Gender**								
Female vs.Male	**3.04**	**1.11**	**8.31**	0.030*	1.62	0.93	2.84	0.092
**Age**								
18–24	2.71	0.38	19.32	0.320	1.49	0.58	3.81	0.409
25–44	2.10	0.61	7.16	0.239	0.97	0.52	1.82	0.925
≥ 45	Ref	-	-	-	Ref	-	-	-
**BMI, Kg/m2**								
≤ 18.5	0.92	0.10	8.91	0.942	1.66	0.52	5.31	0.395
18.5–24.9	0.57	0.21	1.59	0.285	1.54	0.88	2.70	0.130
≥ 25.0	Ref	-	-	-	Ref	-	-	-
**Marital status**								
Single	Ref	-	-	-	Ref	-	-	-
Married	2.02	0.56	7.30	0.283	0.87	0.46	1.64	0.657
Others	0.00	0.00	.	0.998	0.90	0.27	3.05	0.870
**Educational level**								
Below undergraduate	Ref	-	-	-	Ref	-	-	-
Undergraduate	0.61	0.13	2.95	0.542	1.19	0.59	2.37	0.627
Master or above	1.73	0.19	15.43	0.623	**3.27**	**1.14**	**9.42**	0.028*
**Family member infected with COVID-19**								
Yes vs.No	1.78	0.67	4.78	0.250	0.91	0.51	1.61	0.744

Note: *, *p*-value<0.05; **, *p*-value<0.01; ***, *p*-value<0.001.

**Table 5:** Regression models examining the effect of sociodemographic and clinical characteristics on PTSD and depressive symptoms

	PTSD symptoms				Depressive symptoms			
	OR	**95%CI**		p-value	OR	**95%CI**		p-value
		***Lower***	***Upper***			***Lower***	***Upper***	
**Clinical characteristics** ^**#**^								
**Symptoms at onset**								
Sore throat	0.67	0.16	2.76	0.583	0.97	0.48	1.93	0.924
Dry throat	1.01	0.28	3.71	0.987	1.01	0.53	1.92	0.979
Cough	0.64	0.19	2.14	0.464	1.24	0.68	2.28	0.487
Dyspnea	**5.83**	**1.11**	**30.77**	0.038*	1.45	0.52	4.05	0.482
Fever	0.44	0.08	2.28	0.326	0.92	0.43	1.96	0.828
Fatigue	0.40	0.07	2.36	0.309	1.15	0.52	2.54	0.722
Dizziness or Headache	0.54	0.10	2.89	0.471	**2.46**	**1.15**	**5.28**	0.021*
Gastrointestinal symptoms	**6.38**	**1.32**	**30.85**	0.021*	1.91	0.80	4.54	0.144
Loss of appetite	4.13	0.83	20.56	0.083	1.70	0.80	3.61	0.167
Others	1.10	0.29	4.12	0.891	0.71	0.36	1.42	0.336
**Underlying disease**^**#**^								
Yes vs.No	1.85	0.40	8.51	0.432	1.38	0.60	3.17	0.448

Note: Estimates of effect were derived from multivariable logistic regression on PTSD /depressive symptoms with all the variables included

#Adjusted for socio-demographic variables. *, *p*-value<0.05; **, *p*-value<0.01; ***, *p*-value<0.001

## Discussion

In early March 2022, the spike of SARS-CoV-2, Omicron variant, struck Shanghai of China influencing millions of individuals and caused a prolonged lock down without date, which made huge impact on majority populations. The present research firstly estimated the psychological impact of this period on those patients confirmed COVID-19 disease in Shanghai Fangcang shelters. The assessed symptoms encompass PTSS, depressive symptoms, and other questions pertaining to COVID-19. Our study aims to investigate the psychopathological impact of COVID-19 among patients in Fangcang shelters, especially examining the prevalence of PTSS and depressive symptoms, as well as exploring the comorbidity between the two disorders.

### PTSS and depressive symptoms in COVID-19

Although the relationship between PTSD and depression has been discussed frequently in peer-reviewed literatures, little has been done on COVID-19 patients in shelters. In this regard, we reported the psychopathological states of patients in Fangcang shelters timely after the new type of coronavirus, Omicron, overwhelmed Shanghai. Nearly 6% of patients experienced PTSS and over 30% of them exhibiting depressive symptoms. It indicated that patients confirmed COVID-19 were suffering from poor mental health during their treatment and the comorbidity is common to take place. The prevalence of high PTSS in the current study was lower than our previous studies. In the study conducted in Wuhan, the hardest-hit areas in China during the early stage of the pandemic, 7% of the participants met the criteria of PTSS [22].According to the data from a study by Zhu et al., moderate to severe depression and PTSS during the pandemic in US adults was present in 26.7% and 21.8% of individuals, respectively [23].Besides, in another cross-sectional study on patients with COVID-19 one year after being discharged, the prevalence of depression and PTSD were respectively 20.9% and 24.3% [24]. The prevalence of depression was comparable, while the incidence of PTSS was significantly higher compared to our study findings. Apart from the disparity in measurement tools, there may be several other reasons accounting for the discrepancy. Firstly, the mortality rate and severity rate of Omicron were much lower in contrast to early period of the pandemic and majority of the cases transferred to the shelter were asymptomatic [25, 26]. Thus, the disease burden as well as the mental distress were moderated to some extent. On the other hand, high infectivity and concealment of Omicron variant lead to numerous confirmed cases, increasing the difficulties in timely detection and transport [3]. Therefore, the underlying PTSS of some patients may probably mitigate by the time they were transferred to the shelters.

### Risk factors for PTSS

We found that female COVID-19 patients, as well as patients having family members infected had a higher incidence of PTSS, which was in line with the previous studies conducted both in H1N1 epidemic [27]and COVID − 19 [28]. Several studies have proved that women were more likely to develop PTSD as well as other psychiatry disorders than men after traumatic events [29, 30]. After controlling the demographic factors, the multiple logistic regression showed that suffering from dyspnea or gastrointestinal (GI) symptoms were risk factors for PTSS. Recently, increasing GI symptoms and distress was demonstrated to have predictive validity of associations with worse mental health [31]. In a meta-analysis examining the associations between physical distress and PTSS conducted by Pacella et al., it was found that PTSS was related to a higher frequency and severity of respiratory and GI symptoms, which coincides with our results [32]. Additionally, in a Crohn’s Disease sample, GI symptoms were 4-fold worse in patients who met criteria for possible PTSD [33].

### Risk factors for depressive symptoms

Actually, the association between COVID-19 related symptoms and psychological symptoms has not been systematically studied [34].Patients suffering from somatic symptom (sore throat, cough, fever, fatigue, dizziness or headache, gastrointestinal symptoms, loss of appetite) reported more depressive symptoms in our study, which matches the earlier findings indicating that exposure to an increased number of COVID-19 symptoms may be associated with depressive symptoms [35]. In the investigation conducted by Kim et al. in Korean, the presence of COVID-19 symptoms(such as headache/dizziness, sore throat ) at baseline exhibited a significant negative impact on depression at admission and 1 week after hospitalization [34]. According to the data from Poland by Mazurek et al., 65% of all the participants experienced headache during the pandemic; individuals reported headache during the acute infection appeared to have an elevated risk of depressive symptoms [36]. However, since headaches and other physical distress can act as chronic stressors in the development of depression, cross-sectional studies cannot exclude the possibility that individuals currently experiencing depression may be more likely to recall or report headaches.

### Comorbidity between PTSS and depressive symptoms

As expected, patients with PTSS shared high co-occurrence rate with those had depressive symptoms, which was in accord with the previous studies. Earlier examination of comorbidity varied wildly from 4 to 51% according to the DSM-III [13]. According to a recent systematic review across 57 studies, over half (52%) of the participants with PTSD also met the clinical criteria of major depressive disorder [37]. The correlation analysis of the PHQ-9 and PCL-5 scores revealed significant associations of depression and PTSS. The score of PHQ-9 was significantly positively correlated with the total score and the scores of each dimension of PCL-5. Although, wealth of research reported the comorbidity between the two disorders, the interpretation of underlying mechanism remains vague. Previous studies have investigated common risk factors associated with comorbid PTSD and depressive disorder, including female gender, lower educational attainment, history of childhood abuse, and limited social support [38, 39]. There are several hypotheses explaining the relationship between PTSD and depression especially their co-occurrence. For example, the hypothesis promoted that overlap symptoms could account for the comorbidity between PTSD and depression, which attributed to indistinct definition boundaries [40]. However, the rate of comorbidity changes substantively among the different diagnosed versions, which indicate the partial overlap of diagnose criteria is an artifact of comorbidity [40, 41]. Secondly, the two disorders showed in-equivalent bidirectional risk for one another [40]. In view of this opinion, one presumed a causal relationship between the disorders that PTSD may be a risk factor for depression, or vice versa, which could explain the comorbidity. Specifically, in the review of Stander and colleagues, the assumption that PTSD increased the development of depression were successfully supported, while few of evidence suggested that depression can be considered as a risk factor for combat trauma [42]. To some extent, it provided theoretical foundation for the current study. In our study, we found that the comorbidity rate of PTSD and depressive symptoms increased with the severity of depressive symptoms. Further, another proposed that the comorbidity based on a shared vulnerability or risk factors, such as the environmental or genetic factors [43].

### Limitations

Several potential limitations should be acknowledged regarding to the findings of this study. Firstly, self-report measurements used to assess PTSD may be susceptible to bias, particularly as older adults may not have adequate internet proficiency. Additionally, it included a small number of participants and the response rate was low to some extent. Thirdly, the prevalence of PTSD was estimated using an online survey rather than a clinical interview. While online surveys are currently necessary for epidemic prevention purposes, future studies could employ more rigorous and valid measures.

## Conclusions

Overall, despite the inherent limitations of self-report measures and the potential influence of social stigma on a subset of patients, our findings offer a depiction of the mental health status among individuals in the Fangcang shelters. Firstly, the prevalence of PTSS and depression were 6% and 30.1% respectively among the patients. Secondly, majority population who had PTSS also suffered from depression (85%) at the same time. Moreover, prevalence of depression is higher in those with PTSS, and the comorbidity rate increased with the severity of depressive symptoms. The comorbidity was valuable for understanding the mechanism of these disorders in patients and for identifying potential interventions. We propose that the findings might be useful to gain an insight into the assessment of specific individual during the pandemic.

## Data Availability

The data are not publicly available due to the necessity to protect data which are still under analysis for further studies, but can be accessed from the corresponding author if there are reasonable requests.
